# Ammonia threshold for inhibition of anaerobic digestion of thin stillage and the importance of organic loading rate

**DOI:** 10.1111/1751-7915.12330

**Published:** 2015-12-21

**Authors:** Jan Moestedt, Bettina Müller, Maria Westerholm, Anna Schnürer

**Affiliations:** ^1^ Department of Biogas R&DTekniska verken i Linköping ABBox 1500LinköpingSE‐581 15Sweden; ^2^ Department of Microbiology, BioCenterSwedish University of Agricultural SciencesBox 7025UppsalaSE‐750 07Sweden

## Abstract

Biogas production from nitrogen‐rich feedstock results in release of ammonia (NH_3_), causing inhibition of the microbial process. The reported threshold ammonia value for stable biogas production varies greatly between studies, probably because of differences in operating conditions. Moreover, it is often difficult to separate the effect of ammonia inhibition from that of organic loading rate (OLR), as these two factors are often interrelated. This study attempted to distinguish the effects of ammonia and OLR by analysis of two laboratory‐scale biogas reactors operating with thin stillage and subjected to an increase in free ammonia (from 0.30 to 1.1 g L^−1^) either by addition of an external nitrogen source (urea) or by increasing the OLR (3.2–6.0 g volatile solids L^−1^ d^−1^). The results showed that ammonia concentration was detrimental for process performance, with the threshold for stability in both processes identified as being about 1 g NH3‐N L ^−1^, irrespective of OLR. Analysis of the methanogenic community showed limited differences between the two reactors on order level and a clear increase in the abundance of *M* *ethanomicrobiales*, particularly *M* *ethanoculleus* sp., in response to increasing ammonia concentration. Further comprehensive molecular analysis revealed that diverse *M* *ethanoculleus* species dominated in the reactors at a given ammonia level at different OLR. The acetogenic community was clearly affected by both ammonia concentration and OLR, suggesting that the volatile fatty acid load in relation to the higher OLR was important for the dynamics of this community.

## Introduction

Biogas formation from organic material proceeds through an array of reactions and requires a complex microbial community performing different interacting metabolic actions. This anaerobic degradation can be divided into four main steps; hydrolysis, acidogenesis, acetogenesis and methanogenesis (Angelidaki *et al*., 2011). During hydrolysis, extracellular enzymes disintegrate complex macromolecules into monomers such as amino acids, fatty acids and sugars. These compounds are used during acidogenesis by fermentative bacteria, and the main products are volatile fatty acids, alcohols, hydrogen and carbon dioxide. During acetogenesis, the longer fatty acids are converted into acetate and hydrogen/carbon dioxide by different syntrophic oxidation reactions. Finally, acetate and hydrogen/carbon dioxide are converted to methane and carbon dioxide by acetotrophic and hydrogenotrophic methanogens respectively (Zinder, 1984). Alternatively, acetate may be oxidized to carbon dioxide and hydrogen by syntrophic acetate oxidation (SAO) coupled with hydrogenotrophic methanogenesis (Zinder and Koch, 1984; Schnürer *et al*., 1999). The different degradation steps proceed in a synchronized manner and decreased activity of one or several microbial groups can severely affect the efficiency and even lead to process failure.

Protein‐rich materials have high bio‐methane potential (BMP) and are thus interesting materials for commercial biogas production (Ek *et al*., 2011; Nordell *et al*., 2013). Unfortunately, high loads of such materials are often correlated with process instability due to the release of ammonia nitrogen (NH_3_‐N) (Chen *et al*., 2008; Rajagopal *et al*., 2013; Yenigün and Demirel, 2013), causing inhibition of the microbial degradation process. Ammonia is released from degradation of amino acids during acidogenesis, and at elevated concentrations it is toxic for different microorganisms, in particular methanogens (Chen *et al*., 2008). The free ammonia nitrogen (FAN) level resulting in inhibition varies, but in both pure cultures and different reactors acetotrophic methanogens have been shown to be more sensitive to elevated FAN levels than hydrogenotropic methanogens (Koster and Lettinga, 1984; Sprott and Patel, 1986; Angelidaki and Ahring, 1993; Hansen *et al*., 1998a). As a consequence of this, SAO processes involving ammonia‐tolerant species have been shown to develop at elevated FAN levels (Westerholm *et al*., 2012; Werner *et al*., 2014). Despite inhibition of acetotrophic methanogens relatively stable operation is thus possible but commonly with reduced methane yield (Schnürer and Nordberg, 2008; Westerholm *et al*., 2012).

For commercial biogas production, it is important to maximize the methane yield, and this is typically achieved by increasing the organic load. In the case of protein‐rich materials, for example thin stillage, this practice can result in very high FAN concentrations, particularly if the feedstock is concentrated in order to maintain long hydraulic retention time (HRT). Biogas plant operators then need to monitor for indications of ammonia inhibition in order to avoid process failure. In such conditions, it can be problematic to separate the effect of increasing FAN from that occurring due to higher load of organic material. These two factors are interlinked, making it difficult to achieve optimal management conditions, particularly as the maximum FAN concentration for steady, efficient anaerobic digestion processes remains somewhat unclear.

The aim of this study was thus to investigate the NH_3_‐N inhibition threshold concentration in a biogas system treating protein‐rich thin stillage, where methane formation mainly proceeded via SAO. The study was conducted in two laboratory‐scale biogas reactors subjected to an increase in ammonia (0.30–1.1 g L^−1^). The effect of organic loading rate (OLR) was discriminated from that of ammonia concentration by using an increase in the organic load to increase the ammonia concentration in one reactor (R_OLR_) and by adding urea to the other reactor (R_UREA_). Process performance was monitored through analyses of chemical and microbiological parameters.

## Results and discussion

### Reactor performance

**Figure 1:**
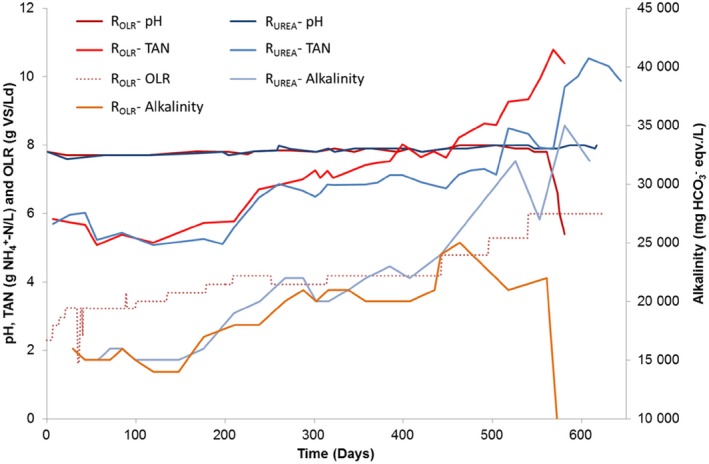
pH, alkalinity and TAN in reactors R_UREA_ and R_OLR_. In addition, OLR is given for R_OLR_. Organic loading rate for R_UREA_ is not shown but was identical to that for R_OLR_ until day 100, and thereafter remained constant at 3.2 g VS L^−1^ day^−1^.

**Figure 2:**
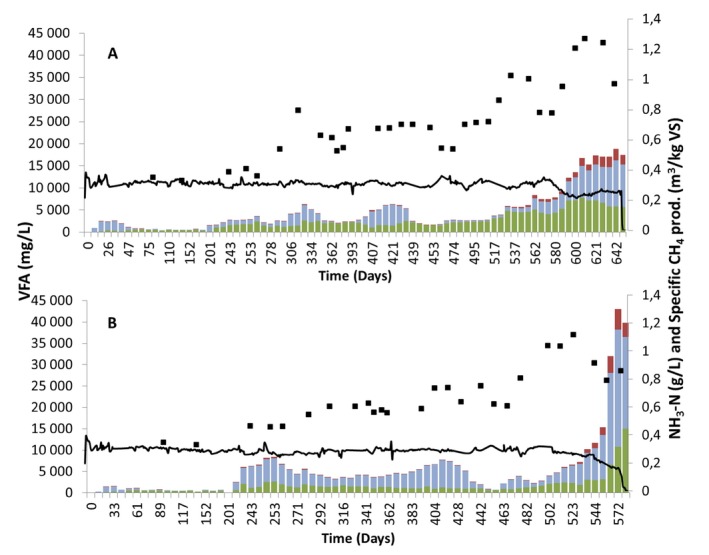
Composition of (acetate ■, propionate ■, residual acids ■), specific methane gas production (—) and FAN concentration (‐■‐) in (A) reactor R_UREA_ and (B) reactor R_OLR_.

The inoculum used in the experiment was obtained from a well‐studied biogas plant (Moestedt *et al*., 2013a, 2013b; Sun *et al*., 2014), treating thin stillage as the main substrate, with an average FAN concentration of about 0.3 g L^−1^ and with steady biogas production for several years. Tracer analysis in previous studies of this biogas plant have confirmed dominance of SAO for acetate degradation (Moestedt *et al*., 2013b; Sun *et al*., 2014). During the start‐up phase (days 1–100), reactors R_UREA_ and R_OLR_ showed similar performance regarding biogas and methane yield as well as levels of ammonia and volatile fatty acid (VFA): 0.32 ± 0.02 L CH_4_ g^−1^ volatile solids (VS), 54.3 ± 0.02% CH_4_, 0.35 g FAN L^−1^, 1.3 ± 0.9 g Tot‐VFA L^−1^ for R_UREA_ and 0.31 ± 0.02 L CH_4_ g^−1^ VS, 53.4 ± 0.02 % CH_4_, 0.35 g FAN L^−1^, 0.8 ± 0.4 g Tot‐VFA L^−1^ for R_OLR_ (Figs 1 and 2). The degree of degradation (on VS basis) was also similar after 100 days with 76 ± 8% and 78 ± 11 % in R_UREA_ and R_OLR_ respectively. The obtained methane yield in the reactors was in line with a previous analysis of the thin stillage, showing a maximum BMP of 0.32 L CH_4_ g^−1^ VS (Moestedt, 2015). The overall performance of the reactors was also similar to those in the commercial biogas plant from which inoculum and substrates were collected, thus indicating a successful start‐up phase, possible to use as a reference period in the study.

From day 100 to day 313, the FAN concentration gradually increased in both reactors from 0.30 to 0.65 g L^−1^ as a result of increasing OLR in R_OLR_ and urea addition in R_UREA_. On reaching 4.2 g VS L^−1^ d^−1^ and a FAN level of 0.46 g L^−1^, which occurred at day 209, the level of VFA increased in R_OLR_. Propionate increased in particular but acetate also accumulated, giving a total VFA concentration of 8.4 g L^−1^ (Fig. 2B). The FAN concentration (0.46 g L^−1^) at that point was at a level previously reported to cause process instability in the reference full‐scale biogas plant (Moestedt *et al*., 2013b). However, by decreasing the organic load to 3.9 g VS L^−1^·day and allowing time for microbial adaptation to the increasing FAN levels, it was possible to reduce the VFA level and achieve process recovery until day 442. During the period between days 100–313, the VFA concentration also increased in R_UREA_ to above 5 g L^−1^, and therefore OLR and urea dose were kept constant between day 313 and day 442 to allow for microbiological adaptation. The total ammonia nitrogen (TAN) concentration in reactors R_UREA_ and R_OLR_ was 6.9 ± 0.13 g L^−1^ and 7.5 ± 0.3 g L^−1^, respectively, and both reactors had FAN concentration of 0.65 ± 0.01 g L^−1^, and the alkalinity in the reactors was 21–22 ± 1 g L^−1^. The specific methane production was also comparable in the two reactors 0.31 ± 0.02 L and 0.29 ± 0.01 L CH_4_ g^−1^ VS, for R_UREA_ and R_OLR_, respectively, close to values from the start‐up phase of the experiment. The pH remained constant during this period in either reactor, and the VFA concentration in R_OLR_ was on average 4.7 ± 0.7 g L^−1^, with 73% consisting of propionate, while in R_UREA_ it was 3.8 ± 0.8 g L^−1^, with 47% as propionate (Figs 1 and 2). The degree of degradation was high, 77 ± 2% and 77 ± 1% in R_UREA_ and R_OLR_ respectively.

After the adaptation period, the OLR/urea dose was again increased. In R_OLR_, the OLR was increased in steps to 4.8 and 5.3 g VS L^−1^ day, finally reaching 6.0 g VS L^−1^ day at day 540. During the same period, the urea dose to R_UREA_ was adjusted to achieve a similar increase in FAN as in R_OLR_ (Table 1). This resulted in a FAN concentration of > 1 g L^−1^ after 500 days in R_OLR_ and 520 days in R_UREA_ (Fig. 2). This high ammonia level caused a rapid increase in VFA in both reactors, followed by a decrease in pH and in specific methane production in the final phase of the process (Fig. 2). This clearly illustrates that the threshold concentration for maintained process stability had been reached.

**Table 1:** Organic loading rate (gVS L^−1^ day^−1^) of reactor R_OLR_ and urea dosage (g d^−1^) of reactor R_UREA_. The OLR of R_UREA_ was constant at 3.2 g VS L^−1^ d^−1^ from day 20. The urea dose was regulated according to the resulting FAN concentration in R_OLR_, and thus the dosage varied somewhat throughout the operation

Days	OLR (g VS L^−1^ day^−1^)	Urea dose (g day^−1^)
0–20	Increase from 2.3 to 3.2	–
21–99	3.2	–
100–134	3.4	0.11
135–178	3.7	0.24
179–208	3.9	0.37
209–251	4.2	0.61–1.2
252–314	3.9	0.48–0.70
315–442	4.2	0.83
443–495	4.8	1.2–1.7
496–539	5.3	1.6–2.3
540–	6.0	2.0–3.6

The fact that the specific methane yield was maintained until the final phase of the experiment reflected successful adaptation of the microbial community to the increased FAN concentration. However, accumulation of VFA occurred periodically, requiring operation at a lower OLR (Fig. 2). Similarly, several previous studies have shown that adaptation of anaerobic digestion to high FAN is possible, most likely as a result of selection of ammonia‐tolerant species. For example, Calli and colleagues (2005) observed process adaptation to FAN concentration above 0.80 g L^−1^ by increasing the level over a long period (450 days) in upflow anaerobic sludge blanket (UASB) reactors treating synthetic wastewater, but still with propionate accumulation of up to 0.5 g L^−1^ (50% of total VFA). In a study in thermophilic continuously stirred tank reactors (CSTR) reactors with HRT of 15 days treating manure, adaptation to reach a FAN concentration of 0.60–0.80 g L^−1^ proved possible, but with higher VFA levels (propionate concentration ∼3.5 g L^−1^) than in low‐ammonia reference reactors (Angelidaki and Ahring, 1994). Another nitrogen adaptation experiment resulted in stable, but lower, methane yield and elevated total VFA concentration (up to 5 g L^−1^) when the FAN concentration was increased from approximately 0.60 to 1.20 g L^−1^ (Angelidaki and Ahring, 1993). Furthermore, both mesophilic and thermophilic commercial‐scale biogas plants (CSTR) have been reported to operate at high FAN levels (0.52–0.80 g L^−1^) for several years with maintained process stability at HRT of 24–60 days (Sun *et al*., 2014).

The process failure in reactors R_UREA_ and R_OLR_ at an almost identical FAN concentration (> 1 g L^−1^) illustrated the low impact of OLR (which at this time was 88% higher than the starting value in R_OLR_) on the critical FAN threshold. The FAN level resulting in process failure was in agreement with values reported previously for FAN‐adapted processes operating at similar temperature and pH (Hansen *et al*., 1998a; Lauterböck *et al*., 2012). However, while process failure in the two reactors occurred at the same FAN level, the breakdown occurred faster in R_OLR_ and the VFA accumulation pattern differed somewhat (Fig. 2). Propionate was the dominant VFA during the whole experimental period in R_OLR_, while in R_UREA_ acetate was the dominant acid up to a FAN level of > 1 g L^−1^, above which the propionate concentration increased rapidly. The difference in VFA accumulation pattern between the reactors can probably be explained by increased throughput of VFAs following the higher OLR in R_OLR_, resulting in higher stress for that microbial community. Since propionate increased in both reactors on reaching the threshold FAN level, it seems to be an indicator of FAN inhibition (Fig. 2), as suggested previously (Angelidaki and Ahring, 1993; Ahring *et al*., 1995). The accumulation of propionate could have been caused either by inhibition of propionate‐oxidizing bacteria or by the degradation in acidogenesis being directed towards more propionate production. Calli and colleagues (2005) reported reduced abundance of acetogenic bacteria in response to increasing FAN level and propionate formation and concluded that propionate formation was caused by inhibition of propionate oxidizers. On the other hand, FAN‐inhibited methanogenesis results in higher hydrogen pressure, which in turn is correlated with a shift in acidogenesis from acetate, hydrogen and carbon dioxide towards production of longer acids (Worm *et al*., 2010). This could also explain the increased production of propionate following higher OLR in reactor R_OLR_.

Reactor R_UREA_ survived without complete process failure for 100 days longer than R_OLR_, but with reduced gas yield compared with start‐up (Fig. 2). The difference between the reactor can be explained by a pH variation (from pH 8.0 to 7.9) and a somewhat lower TAN in R_UREA_, resulting in a reduction of the FAN concentration to 0.77 g L^−1^ due to a shift in equilibrium between NH_4_^+^ and NH_3_ after reaching the FAN threshold. In the present case, the longer process survival in R_UREA_ despite the high VFA concentration could also be an effect of the very high alkalinity, as a result of the high amount of added urea. However, R_UREA_ eventually ceased to produce methane too, at FAN levels above 1.2 g L^−1^.

### Methanogenic community analysis

**Figure 3:**
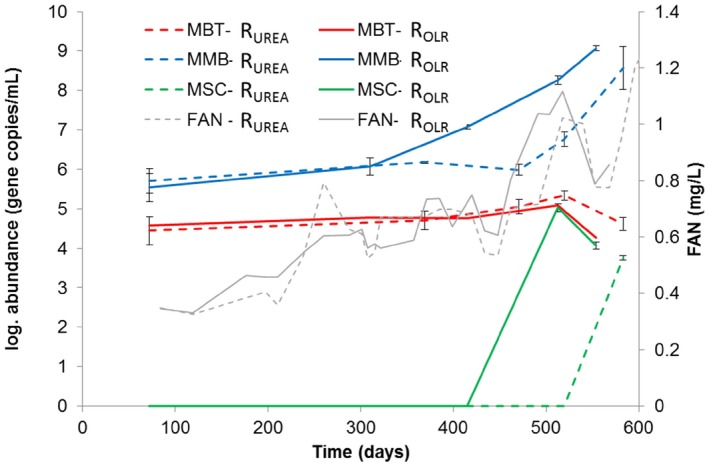
Log abundance of methanogenic groups and free ammonia nitrogen (FAN) concentration in reactors R_OLR_ and R_UREA_ (dotted lines). *Methanobacteriales* (MBT), *Methanomicrobiales* (MMB), *Methanosarcinaceae* (MSC).

**Figure 4:**
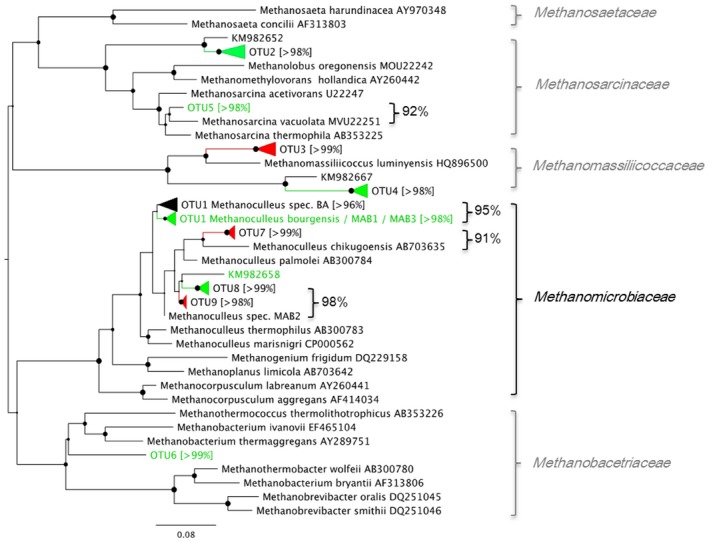
Phylogenetic tree constructed of sequences retrieved in the clone library of the *mcrA* gene. Operational taxonomic units retrieved from three different samples (start‐up and end point of each reactor), with theoretical T‐RF sequence lengths indicated in brackets. Green font indicates dominance in R_UREA_ at end point, red indicates dominance in R_OLR_ at end point, and black indicates similar abundance in both reactors at end point or throughout the experiment. Sequence identity is given (%) for selected genotypes.

**Figure 5:**
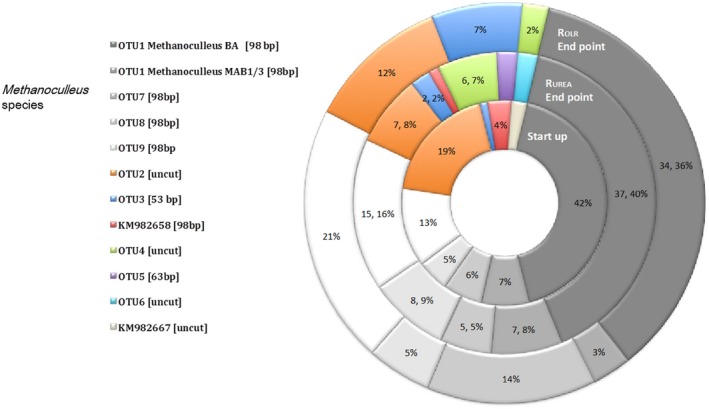
Deeper analysis of the clone library, correlated to the T‐RFLP analysis of the *mcrA* gene. Start‐up represents reactor R_OLR_ at day 72 before any changes were made in the experiment. End point represents day 554 in R_OLR_ and day 583 in R_UREA_.

Quantitative polymerase chain reaction (qPCR) analyses of reactor liquid showed similar levels of hydrogenotrophic *Methanomicrobiales* and *Methanobacteriales* in both reactors (Fig. 3). No *Methanosaetae* or *Methanosarcina* were detected at start‐up, as also reported for other SAO‐dominated processes (Karakashev *et al*., 2006; Sun *et al*., 2014). The absence of *Methanosaetae* was expected due to its apparent sensitivity to high FAN (Sprott and Patel, 1986), as was the dominance of hydrogenotrophic methanogens, which are known to have higher tolerance (Hansen *et al*., 1998a; Angenent *et al*., 2002; Westerholm *et al*., 2011a; Sun *et al*., 2014). With increasing FAN concentration (from 0.30 g L^−1^ to > 1.0 g L^−1^), the abundance of *Methanomicrobiales* increased in both reactors, from log 5.5–5.7 gene copies mL^−1^ during start‐up to log 8.6–9.1 gene copies/ml at the end point, just before process failure occurred. The increase was statistically significant (*P* = 0.001 for R_UREA_, *P* = 0.0001 for R_OLR_; Student's *t*‐test) (Fig. 3). The abundance of *Methanomicrobiales* increased somewhat earlier in R_OLR_ than in R_UREA_ (day 313 onward), which correlated with the earlier increase of FAN in R_OLR_ (Fig. 3). The increase of *Methanomicrobiales* in R_UREA_ was somewhat unexpected and shows that the higher substrate load was not the reasons for the higher abundance. As there was no increase in methane production at the end of the experiment one possible explanation might be that a population shift occurred within the *Methanomicrobiales* towards species harbouring multiple copies of the 16s RNA gene or expressing a less efficient methanogenic metabolism. The *mcrA* clone libraries obtained from start and end points indicate such a species change within the genus *Methanoculleus*, in which e.g. OTU1 is less abundant at the end point compared with e.g. OTU9, which abundance increased in both R_UREA_ and R_OLR_ (Fig. 4; Fig. 5).

Terminal restriction fragment length pattern (T‐RFLP) analysis and subsequent clone library targeting the *mcrA* gene, in which R_OLR_ day 72 was assumed to represent the start‐up phase in both reactors since they had been treated identically until then, showed corresponding results as found in the qPCR analysis, i.e. with higher FAN concentration the community was indeed dominated by increasing *Methanomicrobiales* (Fig. S1; Fig. 4). The 94 bp terminal restriction fragment (T‐RF), which dominated during the whole operating period (70–77%), corresponded to the majority (75–90%) of recovered genotypes in the clone library. These genotypes are summarized as operational taxonomic unit (OTUs) 1, 7, 8 and 9, which were found to be identical or closely related to different *Methanoculleus* species (Fig. S1; Fig. 4). In accordance with the higher gene copy number for *Methanomicrobiales* obtained by qPCR, this T‐RF also showed higher abundance in R_OLR_ at day 415 compared with R_UREA_ at day 471 (Fig. S1).

The most abundant genotype in the *mcrA* clone library was OTU1, representing 34–37% at the end point in both reactors. This OTU was > 96% identical to *Methanoculleus bourgensis* strain BA1. This genotype appeared at constant relative abundance from start‐up to end point in both reactors and hence was apparently not affected by the increasing FAN and OLR (Fig. 5). Interestingly, except for this common genotype the clone libraries retrieved from end points showed differences in the *Methanoculleus* population structure between the reactors, specifically the relative abundance of OTU7 and OTU9 (Fig. 4; Fig. 5). Both these OTUs were recovered at higher frequency in R_OLR_, together providing 35% of the methanogenic community recovered compared with 20% in R_UREA_. Operational taxonomic unit 9 was 98% identical to *M*. *bourgensis* strain MAB2, while OTU7 was 91% similar to *Methanoculleus chikugoensis* (Fig. 4). In addition to these highly abundant OTUs, a rather small fraction of the end‐point community (4–8%) was identified as the type strain *M. bourgensis* CB1 and the *M*. *bourgensis* strains MAB1 and MAB3 (all defined as OTU1 with > 94.5% identity). The dominance of *Methanoculleus* species within *Methanomicrobiales* and the increased abundance of *Methanoculleus* sp. concurrently with the increase in FAN concentration appear typical for ammonia‐dominated processes (Angenent *et al*., 2002; Westerholm *et al*., 2011a; 2012; Nikolausz *et al*., 2013; Sun *et al*., 2014). Moreover, several of the identified species have previously been found in high ammonia processes, i.e. *M. bourgensis* strain BA1, MAB1, MAB2 and MAB3 (Schnürer *et al*., 1999) and tentatively also indicated for *M. chikugoensis* (Cardinali‐Rezende *et al*., 2012). This result further illustrates the high ammonia tolerance of these organisms and suggests an important role in SAO.

*Methanosarcinaceae* and *Methanobacteriales* were not retrieved in the clone libraries and did not appear in the T‐RFLP from R_OLR_. The qPCR analyses showed low or not detectable levels of these species in both reactors, indicating low impact of these species on the methanogenesis in these processes. In R_UREA_, genotypes of these groups were however recovered at the end point, indicating that they might still contribute to a smaller extent to hydrogen, and possibly acetate, consumption (Fig. 5, OTU5, OTU6).

At the end point of both R_UREA_ and R_OLR_, an additional group of hydrogenotrophic methanogens appeared, representing about 9–10% of the partial *mcrA* genes obtained in the clone libraries. These clones, designated OTU3 and OTU4 (Fig. 5), were related to *Methanomassiliicoccaceae*. Members of this family have recently been described as obligate hydrogen‐consuming methanogens, reducing methanol and methylamines instead of carbon dioxide (Dridi *et al*., 2012). Thus, an additional methanogenic pathway might contribute to methane production at high OLR, as also recently concluded by Hori and colleagues for thermophilic biogas processes (Borrel *et al*., 2013; Hori *et al*., 2014). Since only a small proportion of OTU3 was recovered from R_UREA_, the appearance could be correlated to a likely higher availability of substrates as a consequence of higher OLR.

### Acetogenic population analysis

**Figure 6:**
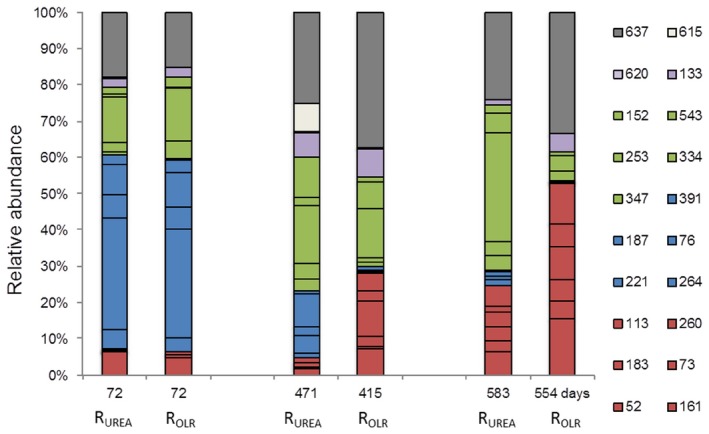
Terminal restriction fragment length pattern analysis of acetogens. Fraction of relative abundance for all sequences with a limit of > 3% relative abundance. Red: enriched in both reactors (but faster and with higher abundance under increasing OLR conditions). Blue: fading in both reactors. Green: increasing under urea dosage conditions (but fading slowly with OLR). Light purple: same behaviour under both conditions. Light brown: single peak. Grey: uncut genotypes.

**Figure 7:**
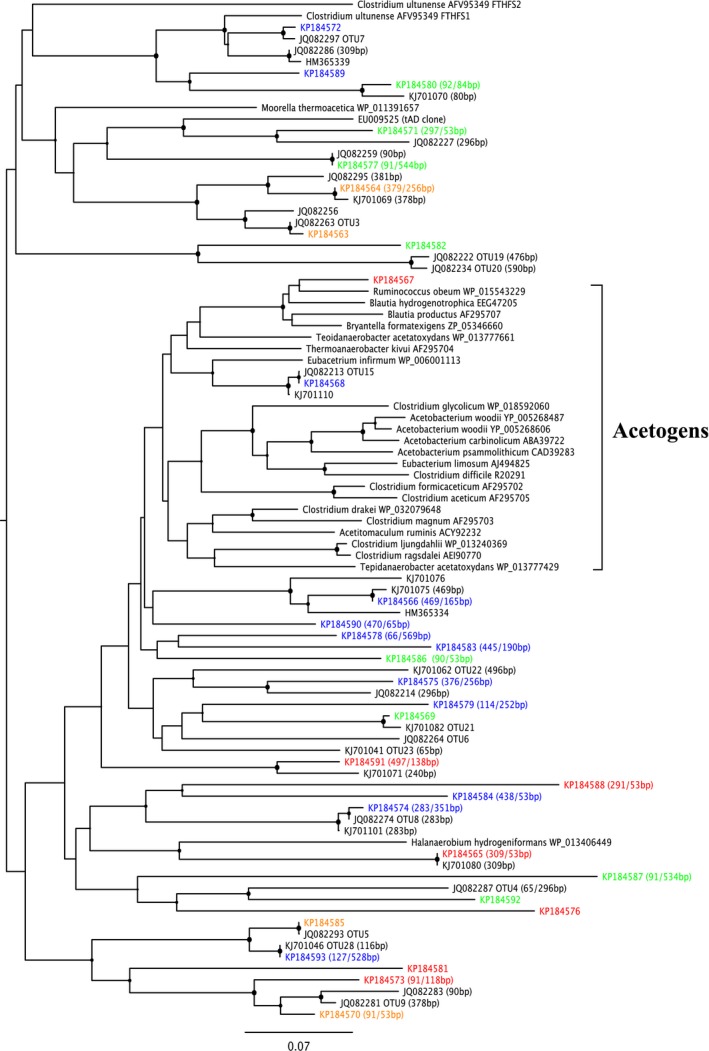
Phylogenetic tree constructed of sequences retrieved in the clone library of the formyltetrahydrofolate synthetase (*fhs*) gene. Operational taxonomic units retrieved from three different samples (start‐up and end point of each reactor). Start‐up sample represents R_OLR_ at day 72 before any changes were made in the experiment. End point represents day 554 in R_OLR_ and day 583 in R_UREA_. Blue group – fading from start‐up; Orange group – detected in both end point clone libraries; Green group – only detected in R_UREA_ end point and Red group – only detected in R_OLR_ end point.

Terminal restriction fragment length pattern analysis targeting the formyltetrahydrofolate synthetase (*fhs*) genes combined with clone libraries was conducted on start‐up and at the end point, i.e. in the same samples as used for analysis of the methanogenic community (Fig. 6; Fig. 7). The T‐RFLP pattern at start‐up was similar for the two reactors and was mainly dominated by T‐RFs 76, 187, 221, 264 and 391 (Fig. 6, blue). However, concurrently with increasing FAN, this initially dominant group clearly declined in abundance in both reactors (Fig. 6, blue). Instead, T‐RFs 52, 73, 113, 161, 183 and 260, which were initially present at low relative abundance, increased at the last sampling point, with a higher presence in R_OLR_ compared with R_UREA_ (Fig. 6, red). Between start‐up and end point, the total group (red) increased from 6% to 15% in R_UREA_ and 5% to 33% in R_OLR_. Within the red group, T‐RF 161 was the dominant T‐RF, and the abundance increased from 4.5% up to 12.3% in R_OLR_, whereas in R_UREA_, this T‐RF had almost the same abundance (5.6%) at the end point as observed at start‐up.

Instead of the red group, which did not increase as much in R_UREA_ as in R_OLR_, another group represented by T‐RFs 152, 253, 334, 347 and 543 increased in R_UREA_ but slowly faded in R_OLR_ (Fig. 6, green group). A fourth group (Fig. 6, purple group), represented by T‐RFs 133 and 620, was present at similar levels in both reactors over the whole operating period, obviously unaffected by increasing FAN and OLR.

**Figure 8:**
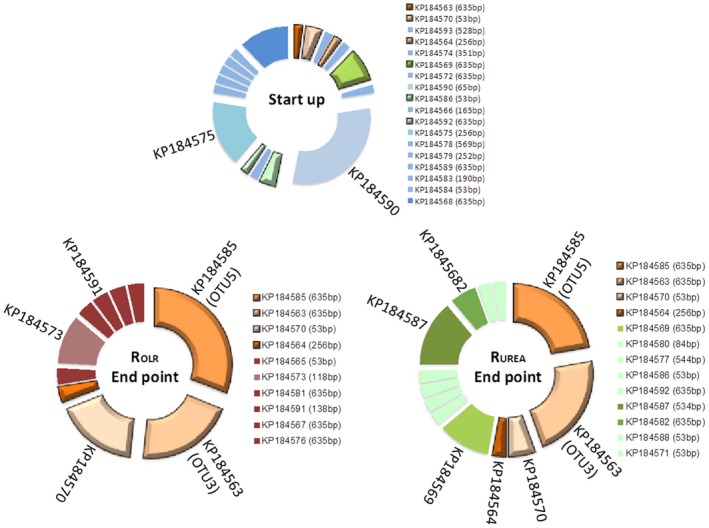
Correlation between T‐RFLP analysis and OTUs identified from clone libraries of the formyltetrahydrofolate synthetase (*fhs*) gene. Start‐up sample represents R_OLR_ at day 72, before any changes were made in the experiment. End point represents day 554 in R_OLR_ and day 583 in R_UREA_. Blue group – Fading from start‐up; Orange group – detected in both end point clone libraries; Green group – only detected in R_UREA_ end point and Red group – only detected in R_OLR_ end point.

The observed changes in the T‐RFLP profiles were also reflected by the composition of the clone libraries. The majority of the genotypes recovered at the start‐up point were not recovered at the end point (Fig. 8, blue group). About 28% and 47% of total number of clones obtained in the libraries were recovered uniquely from R_OLR_ or R_UREA_, respectively (Fig. 8), indicating higher diversity in terms of number of OTUs in R_UREA_. The recovered *fhs* genotypes were mainly distantly related to characterized acetogens (Fig. 7), as previously observed in other biogas systems (Westerholm *et al*., 2011b). On correlating the results from the T‐RFLP analysis with the clone libraries, most T‐RFs could be identified. Among these, clones KP184590 and KP184575 were exclusively recovered from the start‐up point and most likely correspond to T‐RFs 76 and 264, which belonged to the blue group (Fig. 6) declining in both reactors in pace with the increasing ammonia level. In the phylogenetic tree, these clones separated from genotypes recovered from the end point and the higher FAN concentrations (Fig. 7). Despite the comparatively high initial FAN level in reactors R_OLR_ and R_UREA_, KP184590 and KP184575 were closely related or identical to genotypes previously found in low‐FAN systems (Westerholm *et al*., 2011b; B. Müller, *et al*., unpublished).

Genotype KP184570 can probably be considered representative of T‐RF 52 in the red group (Fig. 6) and KP184573 corresponded to T‐RF 113 in the same group. In line with the results from the T‐RFLP analysis, at the end point KP184570 was found at a higher frequency in the clone library of R_OLR_ than of R_UREA_, and KP184573 was exclusively recovered form R_OLR_ (Fig. 8). Clone KP184591, possible corresponding to T‐RF 133 (Fig. 6, purple), was also recovered exclusively from R_OLR_ but at low frequency. However, according to the T‐RFLP analysis, this species appeared at similar but in general low abundances in both reactors in all sample points analysed. The abundance was slightly higher at the end point of R_OLR_ compared with the end point of R_UREA_, which probably explains why it was only recovered from R_OLR_. Thus, this species appeared rather stable and unaffected by both FAN and OLR dynamics in R_OLR_ and R_UREA_.

The green T‐RF group (Fig. 6) was represented by genotype KP184564, most likely corresponding to T‐RF 253. This genotype was present in equal relative abundance in the clone libraries from the end point in both reactors and clustered in the phylogenetic tree (Fig. 7), together with clones previously recovered from a high FAN process (B. Müller, *et al*., unpublished). Another genotype belonging to the green group in the T‐RFLP analysis was KP184587, corresponding to T‐RF 543. This genotype grouped together with two additional genotypes recovered from R_UREA_ (KP184592 and KP184569) and one from R_OLR_ (KP184576) and were related to OTUs previously found to correlate with high ammonia processes (B. Müller, *et al*., unpublished). With a few exceptions, genotypes recovered exclusively from R_UREA_ (Fig. 7, green) did not group together with genotypes recovered exclusively from R_OLR_ (Fig. 7, red).

**Figure 9:**
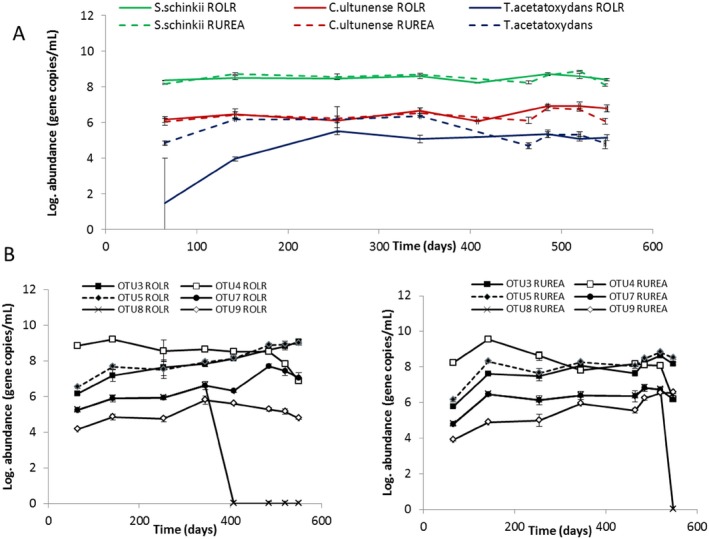
Log abundance of known SAOB (A) and OTUs representing potential SAOB for R_OLR_ and R_UREA_ (B).

Between 13% and 27% of the relative abundance in both reactors (grey bars) remained uncut in the T‐RFLP, this was due to lack of restriction sites (Fig. 6). From the clone library, the two most abundant genotypes in both reactor end points were represented within these uncut T‐RFs. These two members (KP184563 and KP184585; orange in Fig. 8) were identical to OTUs found in a number of high‐ammonia, laboratory‐scale and large‐scale reactors and suggested to be potential candidates for Syntrophic acetate oxidizing bacteria (SAOB) (B. Müller, *et al*., unpublished). In conclusion, the declining blue group (Fig. 6, Fig. 8) in the T‐RFLP was mainly replaced by a core community consisting of KP184585, 63, 70, 64 and possible KP184573, in line with the increase in ammonia level (Fig. 8). In R_OLR_, this core represented 72% of the *fhs* genotypes recovered (exclusive KP184573), compared with 53% in R_UREA_ (Fig. 8, orange), all representing genotypes potentially related to SAO (Fig. 7). Moreover, for both R_OLR_ and R_UREA_, qPCR revealed a stable presence of the SAOB *Tepidanaerobacter acetatoxydans*, *Clostridium ultunense* and *Syntrophaceticus schinkii* (Fig. 9A) and the *fhs* OTUs 3, 4, 5, 7 and 9 (Fig. 9B), which all have been found in biogas processes operating at high FAN levels and which were partly recovered from the clone libraries (*fhs* OTU3 and OTU5). In both processes, the highest log‐scaled gene copy numbers mL^−1^ (8.2–9.0) were achieved for *S. schinkii*, OTU 3 and OTU5 towards the end of the experiment with increasing FAN concentration. The log‐scaled gene copy numbers/ml for *T. acetatoxydans*, *C. ultunese* (5.0–6.9) and OTUs 4, 7 (6.2–7.7) and 9 (4.8–6.6) were equally stable but lower, what likely explains their absence in the clone libraries. It is clear that the SAOB and the major part of the *fhs* OTUs analysed were prevailing in the reactors at these very high FAN levels, a result that support previous studies of high ammonia reactors (Westerholm *et al*., 2011b; Moestedt *et al*., 2014; Sun *et al*., 2014).

Furthermore, the changes observed in the T‐RFLP analyses and clone library following increasing FAN concentration illustrate that the long adaptation period applied allowed enough time for changes in the acetogenic community. It is also quite clear that the T‐RFs and genotypes belonging to the blue group (Fig. 6) were less tolerant to elevated FAN levels than the other groups. The difference of the community structure of the two reactors at elevated FAN could possibly explain the observed difference in VFA profile, with more propionate in R_OLR_.

Terminal restriction fragment length pattern analyses performed on the acetogenic community in a process with increasing FAN concentration (from 0.03 to 0.24 g L^−1^) have previously revealed two distinct shifts in the T‐RFLP profile, indicating that the FAN concentration had a great effect on the composition of the acetogenic community and that an adaptation occurred in response to increasing levels (Westerholm and colleagues (2011b).

Despite the much lower FAN levels than in the present study, a comparable successive effect on the composition of the acetogenic population was observed. One effect observed here for both methanogens and acetogens was that the rise and fall in particular community members appeared to occur faster with the higher VFA throughput in R_OLR_, probably forcing competition between groups able to treat the substrate.

## Concluding remarks

The threshold concentration of FAN to maintain specific methane production in reactors treating thin stillage was determined to be 1.0–1.1 g L^−1^, irrespective of OLR. Regardless of FAN level, the methanogenic community was dominated by hydrogenotrophic *Methanoculleus*‐related species. However, different species within *Methanoculleus* dominated depending on the OLR. A different methanogenic pathway might contribute to methane production at high OLR, as indicated by the recovery of a potential representative from the hydrogenotrophic methanol‐reducing *Methanomassiliicoccaceae*. As regards the acetogenic population, a distinct shift was observed when the FAN level was increased from 0.30 g L^−1^ to the threshold level (1.1 g L^−1^), but the outcome differed depending on OLR. Several of the sequences retrieved from the process at high FAN levels clustered with genotypes previously found in other biogas processes operating at high FAN levels. With increasing OLR, the gene species diversity of the acetogenic population decreased and the composition of the community changed. At the threshold FAN concentration, the propionate level increased irrespective of OLR, indicating that propionate is a good indicator of FAN inhibition. Despite the difference in microbial populations, both reactors displayed process instability at a similar FAN level, suggesting that failure was caused by a general microbial ammonia inhibition threshold rather than limitations caused by overload.

## Experimental procedures

### Experimental set‐up

Two 12‐L CSTR (Tekniska verken i Linköping AB publ.) were operated for 574–653 days (Nordell *et al*., 2011). The active volume was 9 L, and the reactors were agitated continuously at 80 r.p.m. and fed semi‐continuously (once a day) 7 days per week. Volume adjustment and sampling were performed 5 days per week, prior to daily feeding.

Inoculum was collected from a full‐scale reactor (Tekniska verken i Linköping AB, Norrköping) and the OLR of VS during the 100‐day start‐up phase was 2.5 g L^−1^ d^−1^ thin stillage and 0.7 g L^−1^ d^−1^ milled grain. The thin stillage was obtained from a bio‐ethanol plant (Lantmännen Agroetanol AB) in close proximity to the Norrköping biogas plant and frozen (−20°C). The milled grain was supplied by local farmers (Moestedt *et al*., 2013b). During the start‐up period, process parameters and substrates in the laboratory‐scale processes were kept constant in order to mimic the conditions in the full‐scale plant. Thus, temperature and HRT were set to 38°C and 45 days, respectively, and kept constant throughout the experiment. Tap water was used to dilute the thin stillage and the milled grain to reach the HRT of 45 days. After the start‐up phase, from day 100, OLR was gradually increased in reactor R_OLR_. To keep HRT constant, tap water was replaced by thin stillage to obtain the higher OLR, also resulting in a higher ingoing TAN concentration. In the other reactor (R_UREA_) urea (Alfa Aesar GmbH, Germany) was added to the feedstock in order to increase the TAN concentration to the same level as the substrate to R_OLR_, without increasing the loading. The increase in OLR and the urea dose are shown in Table 1. A third reactor (control) was started together with R_OLR_ and R_UREA_ with maintained conditions as used during start‐up throughout the experimental time. This reactor maintained stable process and gas yield and ensured the quality of the results. The daily OLR was calculated as the VS weight fed into the reactor divided by the reactor volume plus feeding volume. A process additive containing iron (11%, a mixture of Fe^2+^/Fe^3+^), cobalt (18 mg kg^−1^) and hydrochloric acid (< 0.5%) was used in both reactors, as in the full‐scale plant (Ejlertsson, 2007; Moestedt *et al*., 2013b). The additive was intended to suppress the H_2_S concentration to 150 μL L^−1^ in the biogas and to keep the cobalt concentration at 0.5 mg L^−1^. Volumetric gas production was measured online with a Ritter milligas counter (MGC‐10, Ritter, Germany) and methane concentration with a gas sensor (BlueSens, Germany). Specific gas production was calculated as the amount of gas produced normalized on amount of organic material (measured as VS) added to the reactor per day. All volumetric gas data presented in this paper have been converted to standard conditions at pressure 1.01325 bar and temperature 273.2 K.

### Chemical analyses

The VFA content was analysed with a Clarus 550 gas chromatograph (Perkin Elmer, USA) with a packed Elite‐FFAP column (Perkin Elmer, USA) for acidic compounds according to Jonsson and Borén (2002). Ammonium nitrogen (TAN; NH_4_‐N) was analysed as the sum of NH_4_–N (aq) + NH_3_–N (aq) by distillation (Kjeltec 8200, FOSS in Scandinavia, Sweden) in an acidic solution (H_3_BO_3_) and NH_4_‐N was then determined by titration with HCl (Titro 809, Metrohm, Switzerland). Kjeldahl‐nitrogen (kjel‐N) was determined with the same procedure and equipment as NH_4_‐N, with the exception that the samples were pre‐treated with H_2_SO_4_ and subsequently heated to 410°C for 1 h. The ammonia (FAN; NH_3_‐N) concentration was calculated from the NH_4_‐N concentration, pH and temperature according to Hansen and colleagues (1998b). pH was measured with a potentiometric pH meter at 25°C using a Hamilton electrode (WTW Inolab, USA). Dry matter (DM) was measured by oven drying at 105°C for 20 h, and VS was subsequently measured by combusting the DM sample at 550°C for 3 h.

### Microbiological analyses

#### Quantitative PCR of methanogenic groups, SAOB and fhs OTUs

##### Methanogenic groups

Samples were withdrawn from the reactors, and deoxyribonucleic acid (DNA) was extracted in triplicate aliquots from each sample according to Moestedt and colleagues (2013a). Five sample points were used for qPCR, chosen to represent equal FAN concentrations in the two reactors. Samples were collected from R_UREA_ on days 72, 369, 471, 520 and 583, and from R_OLR_ on days 72, 310, 415, 513 and 554. Quantification was performed in triplicate using different dilutions (1:10, 1:50, 1:100) to observe and avoid inhibitory effects of the amplification reaction. Analysis of methanogenic groups (*Methanomicrobiales, Methanobacteriales, Methanosaetaceae* and *Methanosarcinaceae*) was performed according to protocols described elsewhere (Westerholm *et al*., 2011a; Sun *et al*., 2014), using IQ SYBR Green Supermix (Biorad, Herculas, CA). *SAOB and fhsOTUs*: Samples were retrieved as triplicates from days 65, 142, 254, 345, 408, 485, 520 and 550 in the case of R_OLR_ and 65, 142, 254, 345, 464, 485, 520 and 548 in the case of R_UREA_, and purified as described in Moestedt and colleagues (2013b). Possible inhibition was excluded by comparing dilution series (1:10, 1:20) of OLR samples prior analyses. Finally, qPCR was performed using 1:20 dilutions according to the protocols described by Westerholm and colleagues (2011a) and (B. Müller, *et al*., unpublished). Melt curve analysis and qPCR data processing were conducted as described previously (Moestedt *et al*., 2013a), DNA was visualized by agarose gel‐red Nucleic Acid (Biotium) electrophoresis using 1.5% agarose gels and 1 kb DNA ladder of Fermentas.

### T‐RFLP

Terminal restriction fragment length pattern analysis of the acetogenic and methanogenic communities was performed on triplicate sampling points, on days 72, 471 and 583 for R_UREA_ and days 72, 415 and 554 for R_OLR_. The acetogenic community was recovered by the following touchdown PCR described by Müller and colleagues (2013) slightly modified: initial denaturation at 94°C for 5 min, followed by 11 cycles of denaturation at 94°C for 60 s, annealing at 63°C for 60 s (decreased by 1°C per cycle to 53°C) and elongation at 72°C for 60 s. Amplicons were enriched by 25 additional cycles conducted at 94°C for 60 s, 53°C for 60 s and 72°C for 60 s, finalized by 20 min at 72°C. The reaction consisted of 35 ng genomic DNA, 20 pmol each 3‐SAO *fhs* primer (Müller *et al*., 2013) and PuReTaq Ready‐to‐go PCR beads of GE Healthcare (Buckinghamshire, UK). The 3‐SAO *fhs* reverse primer (Müller *et al*., 2013) was labelled with 6‐carboxyfluorescein (FAM). To reduce background noise, the respective bands at approximately 630 bp were gel purified using the QIAquick Gel Extraction Kit (Qiagen, Hilden, Germany) even though no unspecific bands were detectable. Deoxyribonucleic acid was visualized by agarose gel‐red electrophoresis using 2% agarose gels and 1 kb DNA ladder of Fermentas. The methanogenic community was analysed by targeting the *mcrA* gene using the forward primer mlas and the FAM‐labelled reverse primer mcrA‐rev (Steinberg and Regan, 2008). The protocol used was: initial denaturation at 94°C for 3 min, followed by five cycles of denaturation at 94°C for 30 s, annealing at 48°C for 45 s (ramp rate 0.1°C/s) and elongation at 72°C for 30 s. Amplicons were enriched by 30 additional cycles conducted at 94°C for 30 s, 55°C for 45 s and 72°C for 30 s, finalized by 10 min at 72°C.

Terminal restriction fragments were obtained by subsequent digestion of the PCR products obtained, as recommended by the manufacturer, with Hpy188III (Fermentas) and BstNI (Fermentas) for analysis of the acetogenic and methanogenic community respectively. Separation of T‐RFs was performed by Uppsala Genome Center (Sweden) using the MapMarker 1000 (Rox) size standard and an ABI3730XL DNA analyser (Applied Biosystems). The fragment data obtained were analysed with Peak Scanner v1.0 software (Applied Biosystems). The resulting profiles were further evaluated in Excel (Microsoft, USA) setting the threshold value at 0.5% of total peak abundance.

### Construction of clone libraries

In order to identify the dominant T‐RFs, clone libraries were constructed from the three sampling points R_OLR_ day 72, 554 and R_UREA_ day 583 for both the acetogenic and methanogenic community. Polymerase chain reaction was conducted as described above from the triplicate DNA extractions using an unlabelled primer combination. The products were gel purified, pooled and cloned into the pGEMT vector system (Promega, Madison, WI USA) as recommended by the manufacturer. CaCl_2_‐competent *Escherichia coli* JM109 cells (Promega) were transformed by the ligation mixture according to the manufacturer's protocol. Clones with at least 94.5% identity on nucleotide level were considered as one OTU. The BlastP search algorithm (National Library of Medicine) was used to identify the closest reference strain. Representatives from every OTU and single clones were deposited in GenBank with the following accession numbers (Table S1; Table S2). Methanogenic community: OTU1 (R_OLR_554: KM982672‐74, KM982676, KM982678, KM982681‐85; R_OLR_72: KM982655, KM982656, KM982658‐62, KM982664‐66, KM982668‐69, KM982671; R_UREA_583: KM982639‐40, KM982644‐47, KM982650‐51), OTU2 (R_UREA_583: KM982641, KM982643, KM982652; R_OLR_72: KM982654, KM982657, KM982670), OTU3 (R_OLR_554: KM982675, KM982677; R_UREA_583: KM982649, R_OLR_72: KM982663), OTU4 (R_OLR_554: KM982679, R_UREA_583 KM982637‐38), OTU5 (R_UREA_583: KM982653), OTU6 (R_UREA_583: KM982648), R_OLR_72 single clone KM982667. Acetogenic community: R_OLR_72 (KP184564, KP184586, KP184592‐93, KP184568, KP184572, KP184589‐90, KP184566, KP184574‐75, KP184578‐79, KP184583‐84), R_OLR_554 (KP184563, KP184570, KP184565, KP184573, KP184581, KP184591, KP184567, KP184576), R_UREA_583 (KP184569, KP184585, KP184580, KP184577, KP184587‐88, KP184582, KP184571).

### Tree construction

Multiple sequence alignments were performed using mafft v7.017 (Katoh *et al*., 2002) and maximum likelihood trees were constructed using PhyML v3.0 (Guidon and Gascuel, 2003), both implemented in Geneious v6.1.8 (Biomatters. USA) including deduced amino acid sequence of partial *mcr*A respective *fhs* sequences affiliated to the accession numbers mentioned above, as well as reference strains. The *fhs* gene tree includes further partial *fhs*‐gene sequences obtained by B. Müller, and colleagues (unpublished), Westerholm and colleagues (2011b) and Westerholm and colleagues (2015). Accession numbers are given in brackets.

## Supporting information


**Fig. S1.** Terminal restriction fragment length pattern (T‐RFLP) analysis of methanogens targeting the *mcrA* gene, with samples from reactor R_OLR_ and R_UREA_ sampled after 72, 471/415 and 583/584 days of operation. Fraction of relative abundance for all sequences > 1% relative abundance limit.
**Table S1.** Clones retrieved from reactor R_OLR_ at day 72 (ROLR72), assumed to illustrate start‐up in both reactors, and from R_OLR_ at day 554 (R_OLR_554) and R_UREA_ at day 583 (R_UREA_583). Identities are based on nucleotide level. Accession numbers are also given.
**Table S2.** Summary of recovered partial formyltetrahydrofolate synthetase (fhs) genotypes. Clones retrieved from R_OLR_ at day 72 (R_OLR_72) before the actual experiment started and from R_OLR_ at day 554 (R_OLR_ 554) and R_UREA_ at day 583 (R_UREA_ 583). Identities are based on nucleotide level. *In silico* restriction fragments are given both as 5'terminal fragment and 3'terminal fragment in order to compare with previous studies^1^ (B. Müller, *et al*., unpublished;^2^ Westerholm *et al*., 2015).
