# Supplementary figures and images for: Ammonia threshold for inhibition of anaerobic digestion of thin stillage and the importance of organic loading rate
Source: Microb Biotechnol. 2015 Dec 21;9(2):180–94. doi: 10.1111/1751-7915.12330 (PMC4767286; doi:10.1111/1751-7915.12330)

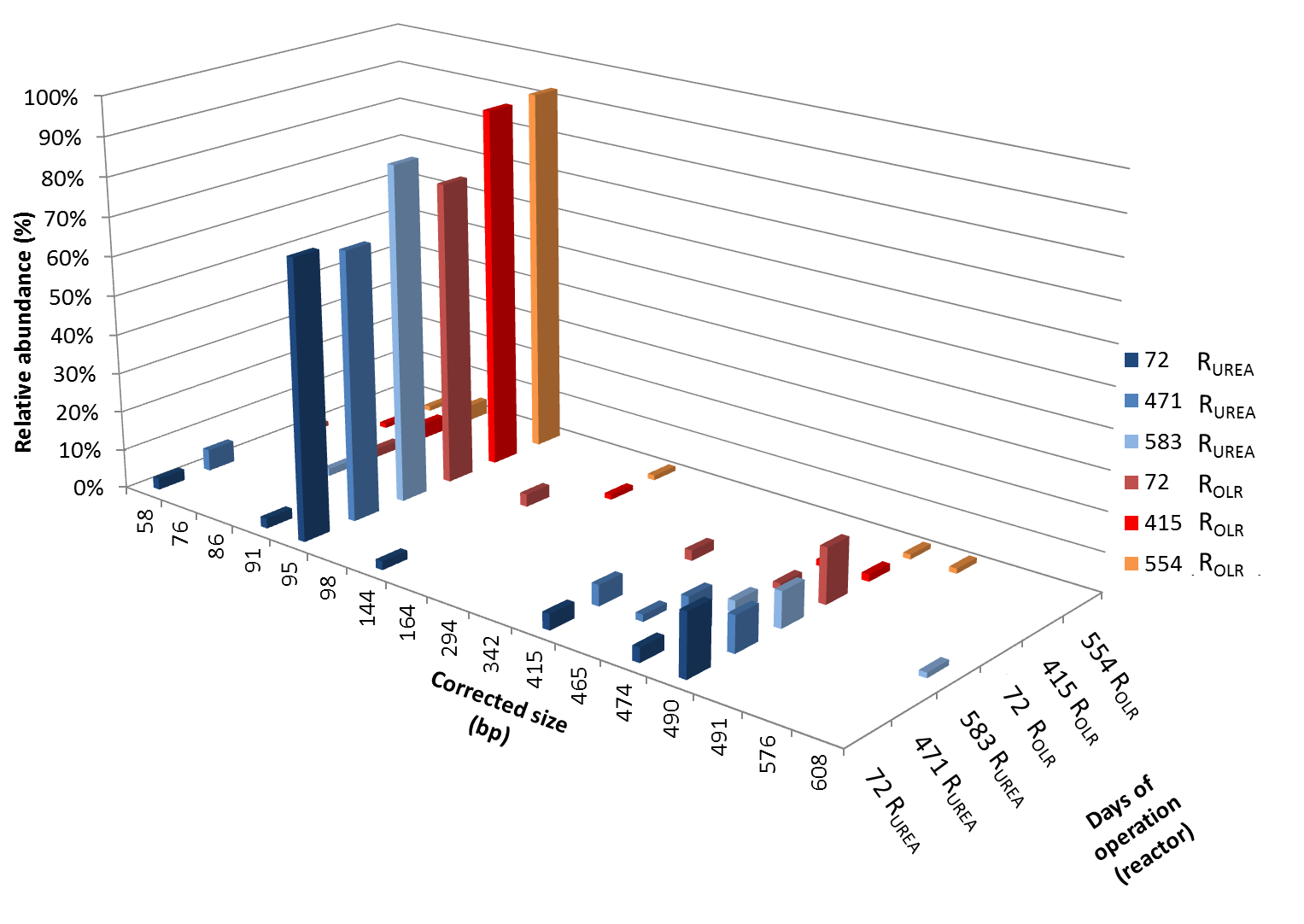

Supplement: Supplementary file 1 — Fig. S1. Terminal restriction fragment length pattern (T‐RFLP) analysis of methanogens targeting the mcrA gene, with samples from reactor ROLR and RUREA sampled after 72, 471/415 and 583/584 days of operation. Fraction of relative abundance for all sequences > 1% relative abundance limit. Table S1. Clones retrieved from reactor ROLR at day 72 (ROLR72), assumed to illustrate start‐up in both reactors, and from ROLR at day 554 (ROLR554) and RUREA at day 583 (RUREA583). Identities are based on nucleotide level. Accession numbers are also given. Table S2. Summary of recovered partial formyltetrahydrofolate synthetase (fhs) genotypes. Clones retrieved from ROLR at day 72 (ROLR72) before the actual experiment started and from ROLR at day 554 (ROLR 554) and RUREA at day 583 (RUREA 583). Identities are based on nucleotide level. In silico restriction fragments are given both as 5'terminal fragment and 3'terminal fragment in order to compare with previous studies1 (B. Müller, et al., unpublished;2 Westerholm et al., 2015). [file MBT2-9-180-s001.zip › MBT2_12330_supp-0001-Figure S1.tif]
